# P-811. Nasal Decolonization with Mupirocin Does Not Diminish the Negative Predictive Value of MRSA Nasal Swab PCR Testing for at Least 7 Days

**DOI:** 10.1093/ofid/ofaf695.1019

**Published:** 2026-01-11

**Authors:** Jeffrey Freiberg, Sharon Ong’uti, Edward Qian, Zhiguo Zhao, Rebecca A Stern

**Affiliations:** Vanderbilt University Medical Center, Nashville, TN; Vanderbilt University Medical Center, Nashville, TN; Vanderbilt University Medical Center, Nashville, TN; Vanderbilt University Medical Center, Nashville, TN; Vanderbilt University Medical Center, Nashville, TN

## Abstract

**Background:**

Detection of Methicillin-resistant *Staphylococcus aureus* (MRSA) colonization using PCR testing of nasal swabs has a high negative predictive value (NPV) for MRSA as the causative organism in community-acquired pneumonia. Accordingly, MRSA nasal swab PCR testing is used clinically to de-escalate anti-MRSA antibiotics. Universal decolonization with intranasal mupirocin is recommended in intensive care units (ICUs) to prevent healthcare-associated MRSA infections. Whether routine mupirocin decolonization impacts the NPV of MRSA nasal swab PCR testing is unclear and represents a major knowledge gap.

**Table 1.**
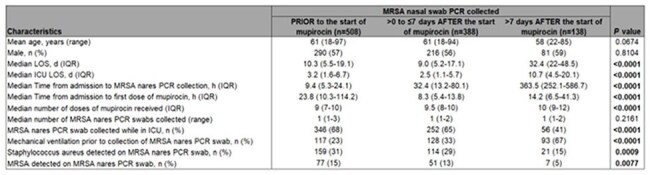
Baseline Patient Characteristics

**Figure 1.**
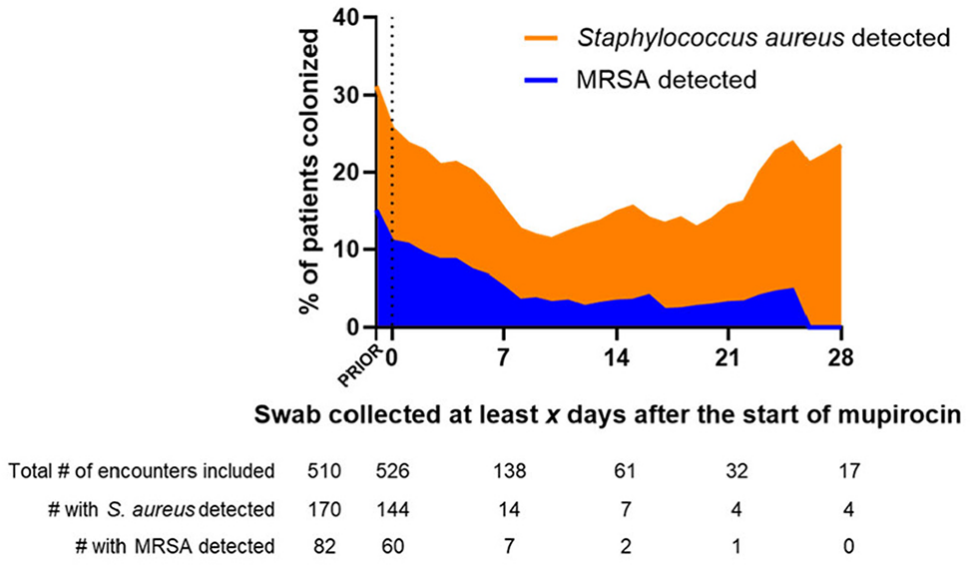
Staphylococcus aureus nasal colonization rates based on time since the start of mupirocin decolonization

**Methods:**

A retrospective, observational study evaluated the association between mupirocin use and the NPV of MRSA nasal swab PCR testing. Adult patients admitted to 8 ICUs across 4 middle Tennessee hospitals who underwent both MRSA PCR testing and started mupirocin decolonization during the same admission were eligible for inclusion if they had any culture collected within 3 days before or 7 days after PCR testing. Patients were grouped based on when they underwent MRSA PCR testing relative to the start of mupirocin decolonization (prior to, 0-7 days after, or >7 days after initiation). The primary outcome was the difference in NPV with a non-inferiority margin of 2%.

**Table 2.**
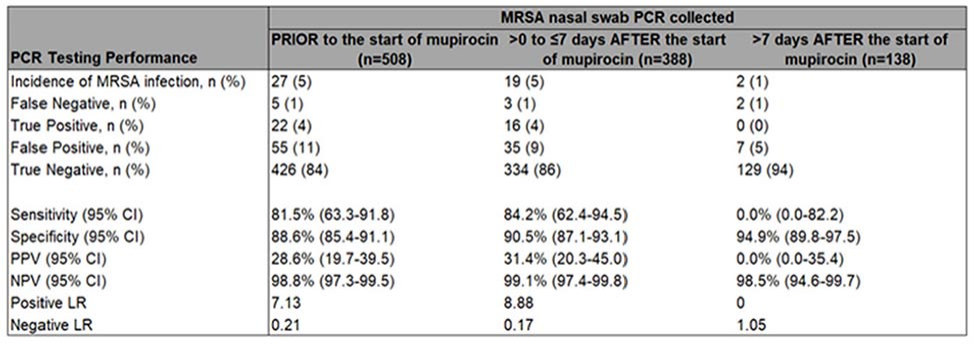
Performance of MRSA Nasal Swab PCR Testing Relative to Timing of Mupirocin Decolonization

**Figure 2.**
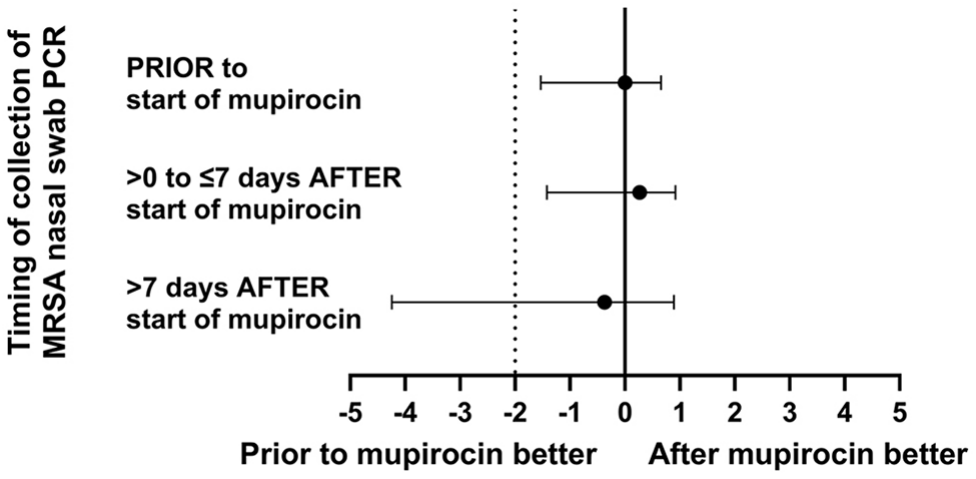
Difference in negative predictive value of MRSA nasal swab PCR testing based on timing of swab collection relative to the start of mupirocin decolonization

**Results:**

We analyzed 1,034 patients who met inclusion criteria from February 2023 to August 2024. Significant differences in baseline patient characteristics were seen between groups (Table 1). MRSA detection by PCR decreased over time following the start of mupirocin decolonization (Figure 1). False negatives were uncommon and the NPV of the test exceeded 98% across all three groups (Table 2). MRSA nasal swab PCR testing in the 7 days following the start of mupirocin decolonization was non-inferior to testing prior to receipt of mupirocin (Figure 2).

**Conclusion:**

MRSA nasal swab PCR testing can be a useful stewardship tool to guide vancomycin de-escalation despite starting mupirocin decolonization. However, due to the very low incidence of MRSA infections following decolonization, its usefulness may be limited beyond one week after mupirocin initiation. These results should allow for expanded use of MRSA PCR testing to de-escalate vancomycin and support decolonization practices to reduce the risk of MRSA infection in ICUs.

**Disclosures:**

All Authors: No reported disclosures

